# Frontiers in Computational Chemistry for Drug Discovery

**DOI:** 10.3390/molecules23112872

**Published:** 2018-11-03

**Authors:** F. Javier Luque

**Affiliations:** Department of Nutrition, Food Sciences and Gastronomy, Faculty of Pharmacy and Food Sciences, Institute of Biomedicine (IBUB) and Institute of Theoretical and Computational Chemistry (IQTCUB), University of Barcelona, Av. Prat de la Riba 171, E-08921 Santa Coloma de Gramenet, Spain; fjluque@ub.edu

Computational methods pervade almost all aspects of drug discovery [1,2,3]. Computer-assisted tools contribute to the decision-making process along the entire drug discovery pipeline, including the validation of suitable targets, high-throughput screening of molecular libraries, the optimization of lead compounds, and the balance between pharmacological potency and physico-chemical and pharmacokinetic properties. This tendency will be reinforced in the next few years due to the continued increases in computer power, and the elaboration of sophisticated algorithms to capture the physico-chemical principles that underlie the activity of drugs. This effort should enable drug discovery methodology to evolve from approximate to more rigorous methods. How should computational methods evolve to ameliorate the success of drug discovery? The answer to this question is related to the identification of the current limitations faced by computational algorithms to unveil the delicate balance between factors that determine both potency and ADMET (absorption, distribution, metabolism, excretion, and toxicology) properties of drug candidates.

This Special Issue aims to provide a forum for the dissemination of recent progress on new computational approaches and methods in the exciting area of drug discovery, primarily concentrated in two reviews, and illustrative examples of application to targets of pharmacological relevance, which are described in eight research contributions.

The first review examines the concept of Dynamic Docking as a promising approach in computational drug discovery [4]. This concept is proposed by Masetti, Cavalli, and coworkers to alleviate the limitations associated with traditional docking simulations, especially regarding the lack of full flexibility in the exploration of the binding between ligand and receptor, as well as the neglect of explicit solvation and entropic effects, which altogether limit the predictive power of these popular, widely used methods. In this contribution, the authors discuss several attempts to introduce Molecular Dynamics concepts into docking simulations, such as solvent mapping calculations, the refining of docking poses via Molecular Dynamics, often coupled with free energy estimates of the binding affinity, the generation of ensembles of protein conformations for docking, and full dynamic sampling predictions of protein-ligand complexes. From a computational viewpoint, Dynamic Docking is analysed through a discussion of advances in ‘biased’ and ‘unbiased’ sampling strategies, as well as progress in the evaluation of methods for checking the reliability of fragment and ligand poses.

Protein-protein interactions (PPI) are the subject of the second review [5]. With an estimated 650,000 PPIs as part of the human interactome, these complexes are generally challenging for drug discovery due to the presence of ‘undruggable’ binding interfaces. In this work Kang, Choi and coworkers examine the structural features of PPI interfaces, highlighting challenging questions that underlie these interactions, such as the elucidation of binding epitopes and hot spots, and the determination of binding site flexibility. Next, they analyse the chemical features of known PPI modulators, and finally attention is paid to emerging in silico approaches for PPI drug discovery, exploiting resources useful for the identification of PPI network topologies, the application of machine-learning algorithms, the application of tools for macromolecular docking and virtual screening, and the disclosure of structural and functional issues with Molecular Dynamics. The review concludes by presenting a prospective analysis of the impact of in silico methodologies in the field of computer-aided PPI drug discovery.

Among the research contributions, Dehez and coworkers examine the problem of determining absolute binding affinities [6]. In this study, this aim is discussed for the binding of netropsin, a well-characterized antiviral and antimicrobial drug, to the minor groove of the DNA double helix. To this end, they employ all-atom Molecular Dynamics simulations and the adaptive biasing force algorithm to compute the standard binding free energy. The system was chosen due to the availability of experimental data that show the preference of netropsin to bind an alternating poly(dAdT)-poly(dAdT) polymer. To explore the limits of the computational strategy, they introduce a series of geometric restraints, and the accurate evaluation of the loss of entropy arising from these restraints is explored by means of PMF (potential of mean force) calculations along tailored collective variables. This strategy allows for an analysis of the different components to the standard binding free energy, paving the way for the extension to ligand binding to macromolecular targets. 

Target selectivity is the underlying topic in the work by Gutiérrez-de-Terán and coworkers [7]. Their contribution is focused on the four G protein-coupled receptors (GPCRs) that signal for adenosine: A1, A2A, A2B, and A3 ARs. Encompassing more than 30% of marketed drugs, the study of GPCRs has been challenged by the limited number of solved structures, which has prevented the application of target-based drug design approaches. Here, the authors review their results on the design and characterization of ligand targeting each of the four adenosine receptors (AR), and further complement these studies with an analysis of the molecular determinants of ligand binding. To this end, the authors present a detailed rationale that relies on the usage of Molecular Dynamics in conjunction with elaborate free energy perturbation protocols. In particular, the authors discuss in silico mutagenesis studies performed to unveil the molecular basis of the specificity and high affinity of the xanthine-antagonist 8-cyclopentyl-1,3-dipropylxanthine to the A1 receptor, the conformational selectivity of a recent series of partial agonists of the A2A receptor, and finally the use of these advanced techniques to the bioisosteric design of ligand targeting the A3 receptor. Overall, this study exemplifies the comprehensive understanding that can be gained from an integrative structure-based drug design against adenosine receptors.

GPCRs are also the subject examined in the contribution by Bojarski, Keserü, and coworkers [8]. In this study, they report a computational protocol to find compounds with selectivity between two receptors: 5-HT_2B_ versus 5-HT_1B_. In contrast to the previous case, a combination of machine-learning methods, docking, and multiple scoring methods is adopted. Machine-learning tools were used to filter a database of drug-like compounds characterized by the Neighbouring Substructures Fingerprint, which is a two-dimensional fingerprint that encompasses information on the connectivity of substructural features of compounds. The docking of selected compounds revealed the preservation of the binding to the orthosteric site, while gaining selectivity through interactions with secondary pockets in the target proteins. Notably, the reliability of this computational approach was supported by the prospective identification of hits with nanomolar affinity and notable selectivity between the two targets.

The study by Tuszynski and coworkers is focused on the structural and dynamical alterations triggered by the G245S mutation in transcription factor p53 [9], which is a protein with mutated variants implicated in more than 50% of human tumours. The majority are missense mutations located in the DNA binding region, and G245S, particularly, is known to be a structural hotspot mutation. To understand the functional implications of this single point mutation, extended Molecular Dynamics simulations were used to explore the differential behaviour between the wild-type and mutant in both apo form and complexed to DNA. The combined analysis of structural changes and per-residue binding energy calculations provide a basis to interpret the destabilization of several structural regions in the protein that are crucial for DNA binding, and the concomitant loss of function associated with the G245S mutation.

Peng and coworkers explore the effect of hydrogen bonding as a determinant of binding in the case of analogues of tricyclic quinoline compound CX-4945 [10], which is the first representative of protein kinase CK2 in human clinical trials. To this end, Molecular Dynamics simulations and residue-based free energy decomposition analysis were exploited to examine the changes in structural and energetic properties arising from the rearrangement of compounds containing non-2,6-naphtyridine substituents due to the lack of hydrogen bonding in the CK2 hinge region. In particular, the results revealed the relationship between defects in the recognition pattern formed by the ligand in the hinge region with the occurrence of distortions in key elements implicated in the coupled conformational mechanism of the enzyme.

With the aim of exploiting the use of photosensitizers in photodynamic therapy, Russo and coworkers address the effect of divalent metal cations (Mg, Zn, Cd, and Pd) on the photophysical properties of tetraphenylporphyrin [11]. From a computational viewpoint, this study required the use of density functional theory and its time-dependent extension to investigate the influence of the metal cations on the electronic structure of these complexes. The study of the low-lying triplet states showed the possibility of all metallotetraphenylporphyrines being able to generate cytotoxic agents. Nevertheless, magnesium- and palladium-containing compounds are proposed to be the best photosensitizer candidates for photodynamic therapy.

Yang, Liu and coworkers propose a simple and effective strategy for the screening of bioactive components of herbal medicines [12], which is exemplified through quantitative pattern-activity relationships (QPAR) on the osteoclastic inhibitory effect of *Herba epimedii*. QPAR quantitatively correlates the chromatographic fingerprint and related bioactivity capacities of samples through several regression methods, leading to the identification of effective bioactive components. Here, the authors resort to joint-action models for modelling the mixture activity based on the Hill equation. In particular, a comparison is made of three joint-action models coupled with Monte Carlo sampling for the screening of bioactive components. A series of active components and compounds in *Herba epimedii* were prepared and validated using the cell assay. Overall, the results support the robustness of joint-action models for unveiling active candidates in the fast screening of natural substances.

Finally, Tarasova and coworkers focus their attention on the prediction of sites susceptible to metabolization in bioactive compounds [13]. Knowledge of the structure of metabolites is relevant for predicting biological activities and toxicities in drug design. This has stimulated the development of different structure-based and ligand-based approaches targeting the prediction of sites of metabolism (SOM), which are reviewed in the first part of this study. Then, the authors examine the reliability of a simple approach for SOM classification that relies on the application of Quantitative Neighbourhoods of Atoms descriptors, which combines information about the connectivity, ionization potential, and electron affinity of atoms, in conjunction with several machine-learning algorithms. The strategy was used for different sets comprising different kinds of chemical modifications, and the results showed that this approach is competitive with other techniques reported in the literature.
